# O03 A fatal case of necrotizing fasciitis due to toxigenic *Corynebacterium ulcerans*: diagnostic surprise with broad public health repercussions

**DOI:** 10.1093/jacamr/dlaf230.003

**Published:** 2025-12-04

**Authors:** Melissa Prior-Ong

**Affiliations:** Microbiology Department, Torbay and South Devon Foundation Trust, Lowes Bridge, Torbay Hospital, Torquay TQ2 7AA, UK

## Abstract

**Background:**

*Corynebacterium ulcerans* is a Gram-positive bacillus capable of producing diphtheria toxin, leading to both respiratory and cutaneous diphtheria. Human infection remains rare in the UK due to high diphtheria vaccination coverage. However, evolving epidemiology—attributed to increasing global travel and domestic pet ownership—has renewed clinical and public health interest in zoonotic and re-emergent toxigenic infections.

**Case presentation:**

An 81-year-old female presented with rapidly progressive necrotizing fasciitis of the right groin, extending from the mid-thigh to the lower abdominal wall. Empirical broad-spectrum antimicrobial therapy with IV meropenem and clindamycin was initiated, and urgent surgical debridement was undertaken. She was admitted to intensive care; however, despite initial stabilization, her condition deteriorated. Two days into her admission, deep tissue cultures revealed *Corynebacterium ulcerans*, which was subsequently confirmed to be toxigenic via PCR detection of the *tox* gene and a positive Elek test. The patient succumbed to her illness at the point of microbiological diagnosis. She had received a full course of childhood diphtheria vaccination and had no recent travel, known zoonotic exposure, or high-risk contacts.

**Discussion:**

This case posed a complex diagnostic and public health challenge, underlining the importance of considering toxigenic cutaneous diphtheria as a rare but potentially fatal cause of necrotizing fasciitis. The absence of classical risk factors and a fully vaccinated status meant that diphtheria was not considered within the differential. Due to the implications of transmission, an emergency multidisciplinary response was mobilized, including infection prevention, microbiology, public health, epidemiology and veterinary services. Risk stratification, contact tracing, chemoprophylaxis for household contacts, and zoonotic source investigation—including screening of household pets—were undertaken promptly.

**Conclusions:**

This case underscores the diagnostic value of early microbiological investigation in severe soft tissue infections and highlights the clinical importance of *C. ulcerans* as an emerging toxigenic pathogen, even in vaccinated individuals. Waning immunity in the elderly may predispose to fulminant disease. Clinicians should maintain an index of suspicion for diphtheria in necrotizing infections. The case exemplifies the critical interface between the microbiology laboratory and frontline clinical decision-making, with immediate and far-reaching infection control and public health consequences.

